# Traditional and low-cost technical approaches for investigating greenhouse gases and particulate matter distribution along an urban-to-rural transect (Greve River Basin, Central Italy)

**DOI:** 10.1007/s10653-025-02456-2

**Published:** 2025-03-27

**Authors:** M. Ferrari, R. Biagi, S. Venturi, F. Frezzi, F. Tassi

**Affiliations:** 1 Dipartimento di Scienze della Terra, Università degli Studi di Firenze, Via G. La Pira 4, 50121 Florence, Italy; 2 Consiglio Nazionale delle Ricerche (CNR), Istituto di Geoscienze e Georisorse (IGG), Florence, Italy; 3 Istituto Nazionale di Geofisica e Vulcanologia, Sezione di Palermo, Via Ugo La Malfa 153, 90146 Palermo, Italy

**Keywords:** Air quality, Low-cost sensors, Greenhouse gases, Particulate matter, Environmental monitoring

## Abstract

Human activities, largely tied to fossil fuels and intensive agriculture, emit massive amounts of climate-altering species and harmful pollutants into the atmosphere that affect soil, ecosystems, and water. Air quality monitoring is crucial to minimize harmful effects and protect human and environmental health. The Greve River basin (Tuscany, central Italy) represents an excellent example of an ecosystem affected by various anthropogenic air contaminants. The upstream areas are predominantly rural, while the downstream zones are characterized by urban and industrial development. Air pollutants throughout the basin were measured adopting two strategies: (i) fixed monitoring at five sites using multiparametric stations equipped with low-cost sensors for CO_2_, CH_4_, and PM_2.5_ concentrations; (ii) measurements along a transect using a mobile monitoring station equipped with a Picarro G2201-*i* analyzer for the determination of CO_2_ and CH_4_ concentrations and ^13^C/^12^C values of the two gases. Results revealed relatively high CO_2_ and CH_4_ concentrations downstream, mainly due to vehicular traffic based on the isotopic signature. The temporal and spatial distribution of the contaminants mirrored the evolution of the Planetary Boundary Layer, with peak concentrations in the early morning due to stable atmospheric conditions, and contaminant dilution due to air turbulence during the daytime. Particulate (PM_2.5_) distribution showed a trend similar to gaseous pollutants, being strongly dependent on wind speed and rainfall events. The high spatiotemporal resolution of data acquisition provided by the low-cost stations for air quality measurements represents an important advance for developing monitoring strategies, complementing the traditional instrumentation commonly used by agencies.

**Supplementary Information:**

The online version contains supplementary material available at 10.1007/s10653-025-02456-2.

## Introduction

Over the past two decades, increasingly stringent community policies aimed at mitigating air pollution emissions have led to an overall improvement in air quality in Europe (European Environment Agency, 2024). Despite these relevant results, air pollution remains the primary environmental and health concern for 90% of the European population (Cavaliere et al., 2018; Feng et al., 2024; Sicard et al., 2021). Urban areas are the most vulnerable to air pollution, exhibiting the highest contaminant levels and the largest impact targets (Fenger, 1999). Although specific pollutants and their impact on health vary across cities worldwide, air contaminants in urban areas frequently exceed both the European Environmental Agency’s (EEA) standards and the World Health Organization’s (WHO) guidelines, leading to air quality issues that diminish life expectancy (Commissione europea, 2021; Energy Agency, 2016). Beyond health, air pollution detrimentally affects vegetation, ecosystems, water, and soil quality, with strong effects on the ecological balance at both local and regional scales. Urban areas are accountable for 80% of the global carbon dioxide (CO_2_) emissions (Oke et al., 2017) and approximately 21% of global anthropogenic methane (CH_4_) emissions (Hopkins et al., 2016; Marcotullio et al., 2013). The latter value was likely underestimated, due to challenges in measuring leaks from gas pipeline networks (e.g., Alvarez et al., 2018; Plant et al., 2019; Ren et al., 2018; Venturi et al., 2021). Moreover, being often situated on plains surrounded by slopes, urban areas experience distinctive weather patterns and atmospheric circulation conditions, such as thermal inversion events, which notably influence air quality. However, air pollution extends beyond city borders: pollutant sources are widespread, some originating from rural areas, and, depending on weather conditions, may extend their impact at a regional and global scale (EEA—European Environment Agency, 2023).

Effective and cost-efficient air quality monitoring is crucial to support the design of policies and actions to circumscribe damage to the environment and safeguard human health, in consideration of the wide variety of factors controlling air pollution: (i) different potential emission sources (e.g., traffic, industries, residential or natural sources), (ii) meteorological conditions acting on pollutant transport, (iii) orography, (iv) chemical processes involving contaminants (Ho, 2012; Vallero, 2014). This complexity requires monitoring systems with high spatial and temporal resolutions, capable of providing insights into pollutant levels across urban and rural settings. The European Union mandates monitoring under Directive 2024/2881 (recent revision of 2008/50/EC), requiring Member States to assess, report, and publish air quality data. The traditional monitoring methods primarily involve stationary stations, whose number and positioning are dictated by population density and the site classification criteria. These measurement stations are commonly equipped with sophisticated, bulky, and expensive instruments that require considerable economic resources for regular maintenance and calibration. This prevents the construction of a high-resolution monitoring network representative of the whole area’s complexity. Two approaches are catching on to address this challenge. The first regards the use of mobile monitoring stations measuring air pollutants at a high spatial resolution over a definite time (e.g., Apte et al., 2017; Desouza et al., 2020). This is an approach increasingly explored as an additional tool for air quality data (Van den Bossche et al., 2015) with several purposes, including assessing personal exposure by equipping the study object with a portable monitor (Dons et al., 2011; Spira-cohen et al., 2010). The main aim is to study spatial variation in air pollution (MacNaughton et al., 2014), or to investigate seasonal and regional variation of the contaminant distribution (Bukowiecki et al., 2003). Mobile monitoring stations record a single snapshot of air pollution within a specific location and time frame, providing a dataset not able to exhaustively depict the temporal variability of air quality in a specific site of interest. Similar to the data gathered using a traditional stationary station, these measurements depend on different parameters, e.g., traffic dynamics, diurnal meteorology, source strength, and site morphology (e.g., Apte et al., 2017; Desouza et al., 2020 and references therein). The second approach is based on the use of a dense network consisting of low-cost stations positioned at fixed points within the study area. Such an alternative stationary method has been pulsed in recent years by the continuous technological development and miniaturization of instruments measuring the concentrations of air contaminants (e.g., Biagi et al., 2024; Clements et al., 2017; Collier-Oxandale et al., 2018; Heimann et al., 2015; Kumar et al., 2015; Popoola et al., 2018; Sun et al., 2016; Van den Bossche et al., 2017).

This study aims to combine these two different monitoring approaches to provide a comprehensive sketch of the air quality in the Greve River basin (GRb; Tuscany, central Italy). This is an area of great socio-economic interest inhabited by about 100,000 people, where rural landscapes reaching 800 m a.s.l. alternate heavily urbanized and industrial areas downhill. This orography, typical of several cities and their surroundings, may strongly influence the air quality through meteorological phenomena of thermal inversion that prevent vertical mixing, keeping air contaminants at the near-ground level and enhancing their impact on human health and overall ecosystems. A network of five low-cost stations (Biagi et al., 2024) was installed within the study area at fixed sites that were identified as potentially affected by diverse sources of pollution to monitor CO_2_, CH_4_, and PM_2.5_ concentrations in the summer and autumn of 2022. Data acquisition strategy was integrated with the measurements obtained from three surveys carried out using a mobile monitoring station equipped with a high-precision instrument for CO_2_ and CH_4_ concentrations, and isotope (^13^C/^12^C) measurements, along a transect of the GRb.

## Study area

**Fig. 1:**
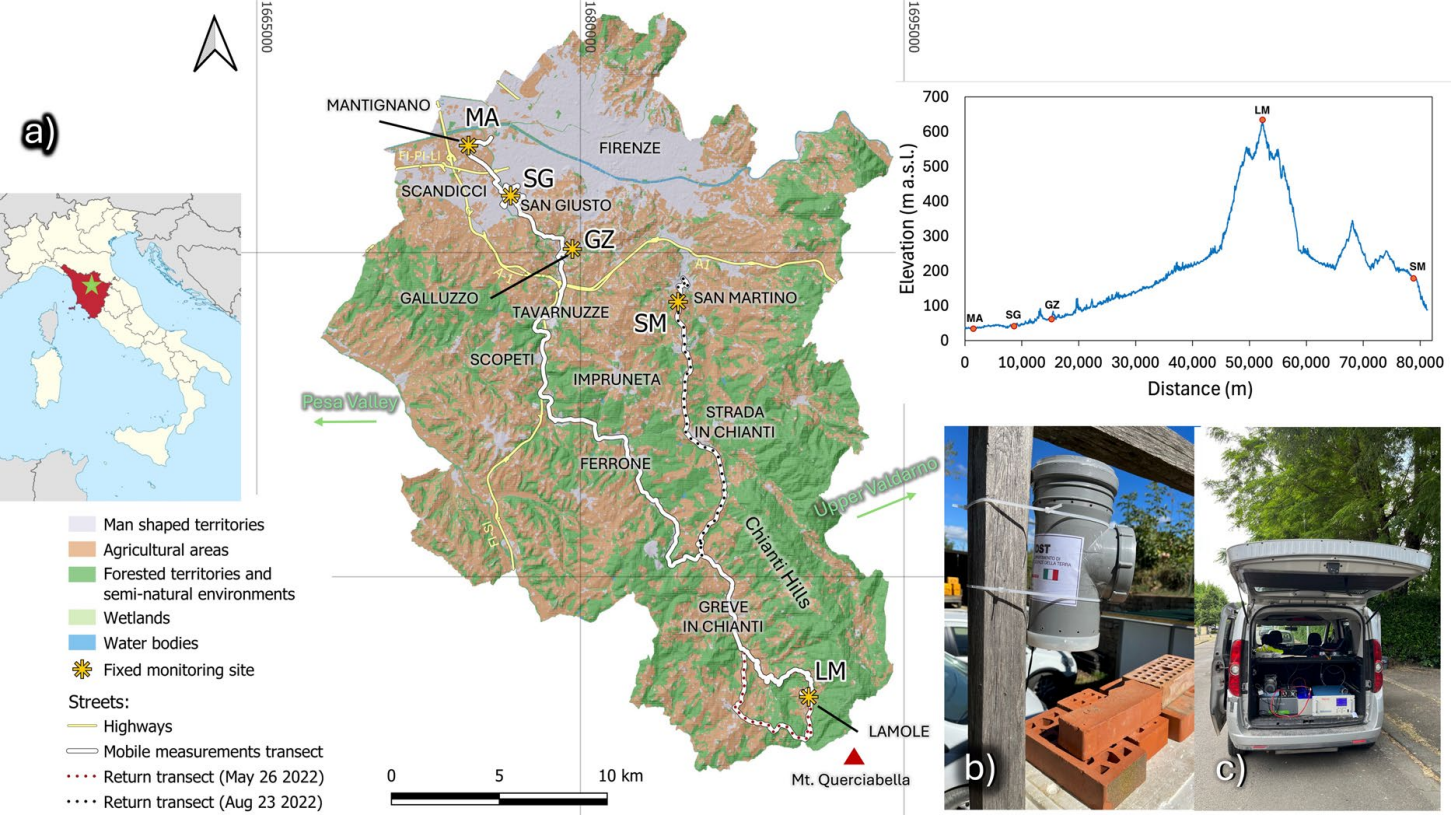
**a** Location of the study area, with marked fixed points (red dots), the mobile track, and the main highways present in the area; **b** low-cost station geometry, **c** mobile station equipped with Picarro G2201-i analyzer

The GRb, south of Florence (Tuscany, Central Italy), represents an ideal site to study and monitor the complex atmospheric pollutant dynamics in a widely urbanized alluvial plain, surrounded by hilly and rural areas devoted to agriculture (Fig. 1a), where different pollutant sources and peculiar weather conditions affect air quality. It belongs to the major basin of the Arno River, being part of the sub-basin of the Middle Valdarno (Cimoli, 2014). The drainage basin (284 km^2^) has a predominantly hilly nature and borders the Pesa Stream to the W and S, and the Strada in Chianti and Impruneta Hills to the E, the latter acting as watersheds with the minor basins of the Upper Valdarno. Mount Querciabella (Municipality of Greve in Chianti) represents the upper part of the GRb, where the Greve River originates at about 800 m a.s.l. In the first stretch, it flows north-westward with a torrential regime on steep slopes dominated by woods, where minor villages and localities devoted to wine production and enology tourism (e.g., the locality of Lamole at c.a. 600 m a.s.l.) are located (Fig. 1a). When reaching the village of Greve in Chianti, the river enters a wide valley flowing through the Chianti Hills Territory (c.a. 300 m a.s.l.), a c.a. 28,000 inhabitants’ rural area (14,390 in the Municipality of Impruneta, and 13,322 in Greve in Chianti; ISTAT, 2023) primarily devoted to agriculture, with a prevalence of olive trees and vineyard cultivation alternated with small woods covering the steepest areas (Fig. 1a). Clay excavation and handicraft activities related to the production of a ceramic material known as *Cotto Imprunetino* occur further downstream, in the village of Il Ferrone (c.a. 130 m a.s.l.) (Fig. 1a). From the locality of Scopeti to Tavarnuzze (c.a. 100 and 75 m a.s.l., respectively), the basin is bordered by the southern foothills of the Monte Albano Ridge, which the river cuts flowing north-eastward close to the highway connecting Florence to Siena. The Greve River receives the waters of the Ema Stream on its right bank at the Charterhouse of Florence, located close to the residential village of Galluzzo (58 m a.s.l.). This represents a hub for Florentine traffic connecting the city center with the A1 Milan-Naples highway and the southern territories (Fig. 1a). The proper urban stretch of the river begins at Scandicci town (c.a. 50 m a.s.l.) and, about 4 km downstream, joins the Arno River on its left bank at the locality of Mantignano, Florence (Cimoli, 2014). In this alluvial plain, manufacturing settlements are highly developed in the urban area of Scandicci (and to a lesser extent in Galluzzo village) where, in addition to industrial activities, land use is largely dedicated to residential areas with continuous and discontinuous fabric (Fig. 1a), hosting a total of about 67,000 inhabitants (ISTAT, 2023).

The GRb experiences a temperate climate marked by mild winters and warm, humid summers. During the latter, air temperature in the urban area downstream often exceeds 40 °C, while the rural areas maintain slightly lower temperatures (Autorità di Bacino del Fiume Arno, 2023). This phenomenon, known as the Urban Heat Island (UHI) effect, commonly occurs in major cities, resulting in metropolitan areas being significantly warmer than their rural surroundings, especially during the hottest months (Petralli et al., 2006). Annual rainfalls in the GRb are 800–900 mm in urban downstream areas and 900–1000 mm upstream, with patterns displaying minimal values in July and peaks in November (Autorità di Bacino del Fiume Arno, 2023). Finally, the prevailing winds in the Plain of Florence blow from the NE-E and SW-W sectors, in the cold and warm seasons respectively.

## Measurement strategy, materials, and methods

### Fixed sites monitoring

Five sites were selected to place stationary multiparametric low-cost stations for air quality monitoring (Biagi et al., 2024) during the summer (from August 23 to September 5) and the autumn (from September 21 to October 11) of 2022. The 5 sites (Fig. 1a) are representative of the differentiated and complex context of the study area, considering the classification criteria provided by the Italian Legislative Decree 155/2010 (transposing the EU Directive 2008/50/EC). This Decree recognizes distinct monitoring sites according to their location (i.e., urban, suburban, and rural areas), and the dominant emission sources (i.e. traffic, industrial, background). The LM site (Lamole, Greve in Chianti), located upstream (c.a. 600 m a.s.l.), was the furthest from the Florentine alluvial plain. It was selected as representative of a rural background environment, where few agricultural activities occur (Fig. 1a). More in detail, the LM site was in a vineyard close to a storage shed of agriculture tools and vehicles mostly used during the grape harvest. The SM site (San Martino, Impruneta), instead, was selected as representative of a suburban background, being located in the backyard of a small parish where olive tree cultivations prevailed but closer to the metropolitan center (Fig. 1a). The GZ site (Galluzzo, Firenze) was selected as a suburban traffic station, being in a residential area connecting the urban center; the monitoring station was installed close to the center square of the village and near schools and surgery, thus representing another area with a vulnerable air pollution target. Finally, the SG (San Giusto, Scandicci) and MA (Mantignano, Florence) sites were in the urban downstream section of GRb and represent traffic and urban background environments, respectively (Fig. 1a).

The multiparametric low-cost instruments (Fig. 1b) were equipped with sensors for the measurement of (i) CO_2_ (Sensirion SCD30), (ii) CH_4_ (Figaro NGM2611-E13), and (iii) particulate matter (PM_2.5_) concentrations (Nova Fitness SDS011), and (iv) air temperature (T) and relative humidity (RH) (Adafruit DHT22; Biagi et al., 2024). The procedure used to calibrate the CO_2_ sensors (in ppm) and convert the voltage output of the CH_4_ sensors in concentration values (in ppm), described in detail by Biagi et al. (2024), involved a supervised machine learning algorithm that provided non-parametric correction models. These were obtained by measurements against a Picarro G2201-*i*, which was used as a high-precision reference of CO_2_ and CH_4_ based on CRDS (Cavity Ring-Down Spectroscopy) (PICARRO INC, 2023). The calibration procedure yielded CO_2_ and CH_4_ concentration measurements with mean absolute errors of less than 4 ppm and 0.03 ppm, respectively (Biagi et al., 2024). Part of the calibration datasets included measurements carried out at the SG (July 4 to 6) and GZ (September 21 to October 11, 2022) sites and was employed for training and testing the machine-learning models used during the calibration procedure (Fig. S1) (Biagi et al., 2024) At GZ, δ^13^C values of CO_2_ and CH_4_ were also measured using the Picarro G2201-*i* analyzer, which was calibrated at the beginning of each measurement period using manufactured standards, as follows: (i) 380, 500, and 1000 ppm for CO_2_; (ii) 1.2, 3.3, and 6.7 ppm for CH_4_; (iii) − 44, − 5, and + 2‰ for δ^13^C-CO_2_, and (iv) − 60, and − 25‰ for δ^13^C-CH_4_ (Air Liquide). The precision of the Picarro instrument was within 0.2 ppm (CO_2_), 0.05 ppm (CH_4_), 0.16‰ (δ^13^C–CO_2_), and 1.15‰ (δ^13^C–CH_4_).

As far as the PM sensors are concerned, previous studies yielded good outcomes with regard to sensor accuracy and sensitivity in outdoor environments (Carotenuto et al., 2020; Cavaliere et al., 2018; Gryech et al., 2020), therefore this work relied on the calibration provided by the manufacturer. To avoid possible errors related to instrumental drift, the repeatability of the sensor signals was evaluated. The results demonstrated that all five sensors yielded analogous and concordant outputs (Fig. S2).

### Meteorological parameters

Temperature and RH were measured by sensors installed in the low-cost stations, while wind direction, wind speed, and precipitation data were downloaded from the database of the Regional Hydrological Service (SIR), accessible online at http://www.sir.toscana.it/consistenza-rete. Two meteorological stations were selected as representative of the upstream rural area (Lamole station—ID: TOS01001115, at 536 m a.s.l), and the downstream urbanized zone (San Giusto station—ID: TOS01001215, at 42 m a.s.l.), respectively.

### Mobile measurements

Concentrations and δ^13^C values of both CO_2_ and CH_4_ in air along a transect within the GRb (Fig. 1a) were measured using the Picarro G2201-*i* analyzer installed on a mobile monitoring station during three surveys, as follows: (i) on May 26, (ii) August 23, and (iii) October 25, 2022. The analyzer, powered by two 12 V–100 Ah batteries, was housed in the back of a car and a 1.5 m long Teflon tube (3 mm diameter) drove air from the top of the vehicle to the inlet port of the instrument (Fig. 1c), through a pump with a sampling rate of 25 mL min^−1^, providing 1 measurement per second (PICARRO INC, 2023).

On May 26, the mobile station moved along a 50 km long route, passing through the urbanized centers and reaching the rural upstream areas (Fig. 1a). Measurements were performed stopping (~ 3 min) at 19 and 11 selected waypoints at the outward (1 point every 2.5 km on average) and at the return (1 point every 4 km on average), respectively, starting at 8:00 and ending at 14:00. Similar measurement routes were carried out during the two following surveys (in August, during the return route, the car stopped at SM to install the multiparametric low-cost station).

## Results

### Fixed sites monitoring

Descriptive statistical parameters (minimum, maximum, mean, median, and standard deviation) computed on 15-min-averaged data measured at each fixed site are reported in Table S1.

**Fig. 2:**
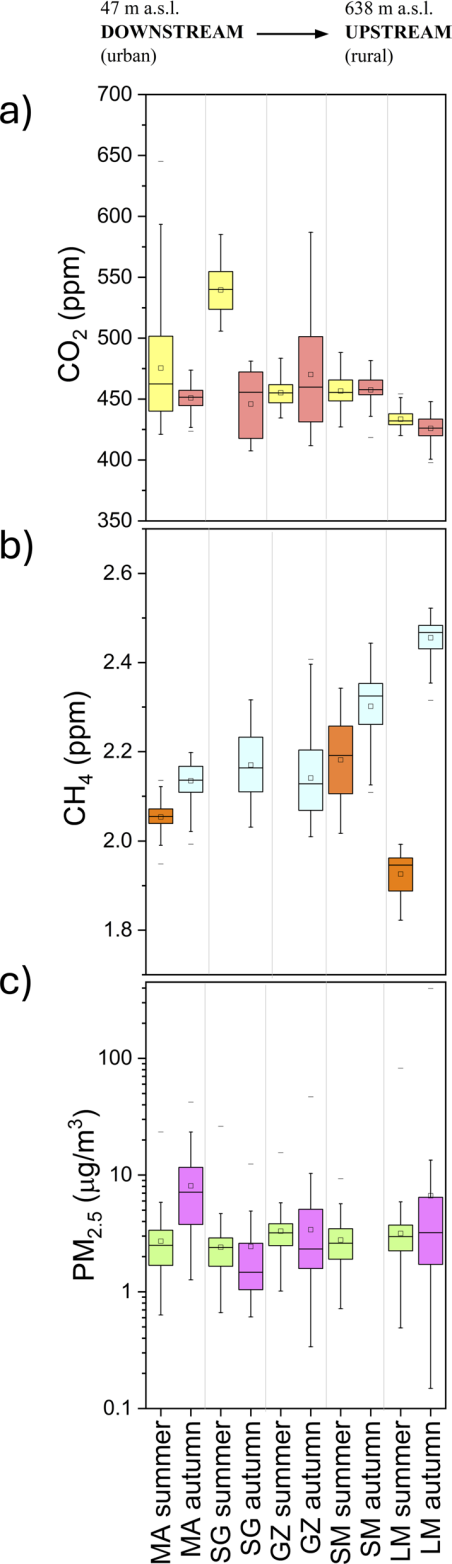
Boxplots of 15-min-averaged (**a**) CO_2_, (**b**) CH_4_, and (**c**) PM_2.5_ concentrations recorded at five fixed stations during the two monitored periods (Summer and Autumn 2022). The small square inside the box represents the mean value, whereas the median value is shown by the black line

During the summer season, the CO_2_ concentrations at the urban sites (MA and SG) ranged from 421 to 645 ppm, and from 506 to 585 ppm, respectively, while at the rural site (LM) they ranged from 420 to 454 ppm. Intermediate concentrations were measured at the suburban background site (SM), where CO_2_ ranged from 427 to 488 ppm, and at the suburban site (GZ), where CO_2_ ranged from 435 to 483 ppm (Fig. 2a). Methane concentrations showed small variations at LM (ranging from 1.82 to 1.99 ppm) and at MA (between 1.95 and 2.14 ppm), while at SM they were in a wider range (from 2.02 to 2.34 ppm) (Fig. 2b). Methane was not measured at SG and GZ sites during the summer monitoring due to a sensor malfunction. The PM_2.5_ showed similar mean values in the five fixed sites (from 2.40 to 3.15 µg/m^3^, Fig. 2c).

During the autumn season, CO_2_ concentrations showed relatively wide ranges at SG and GZ (from 408 to 481 ppm, and from 412 to 587 ppm, respectively; Fig. 2a), while they ranged from 424 to 474 ppm and from 398 to 448 ppm at MA and LM, respectively. Methane concentrations showed the highest values at LM (ranging from 2.32 to 2.52 ppm), while they were significantly lower in the other stations (Fig. 2b). On the contrary, the highest average PM_2.5_ values were measured at the urban site MA (ranging from 1.27 to 42.3 µg/m^3^), while the lowest ones were measured at the SG site (ranging from 0.61 to 12.5 µg/m^3^; Fig. 2c). At the suburban site (GZ), the PM_2.5_ ranged from 0.33 to 46.9 µg/m^3^, and, finally, the widest variation range for this particulate fraction was recorded at the rural LM site, with PM_2.5_ values ranging from 0.15 to 397 µg/m^3^ (Fig. 2c). Due to sensor malfunction, the PM_2.5_ concentrations at SM were not available. During this period, data recorded at the MA and SG sites were lesser than those measured at the other sites, due to a station malfunction.

The CO_2_ and CH_4_ concentrations and their isotopic signature measured by the Picarro analyzer at GZ from September 21 to October 11, 2022, are reported in Table 1 (GZ*, *Type station: High Precision measurements*). Carbon dioxide and CH_4_ showed similar values to those obtained from the low-cost station in the same site (from 410 to 602 ppm, and from 2.00 to 2.45 ppm, respectively). The δ^13^C-CO_2_ values were from − 14.5 to − 8.02‰ versus V-PDB, while those of δ^13^C-CH_4_ were from − 47.7 to − 39.7‰ versus V-PDB (Tab. 1).

**Table 1:** Summary statistics (minimum: Min., maximum: Max., mean, median, and standard deviation: S.D.) of 15-min-averaged CO_2_ and CH_4_ *high precision* concentrations (in ppm) and isotopic compositions (in ‰ vs. V-PDB) at GZ site, measured by Picarro G2201-i analyser

Point	Type station	Monitoring period		CO_2_	CH_4_	∂^13^C-CO_2_	∂^13^C-CH_4_	T	RH
GZ*	Suburban traffic (high precision measurements)	21/09–11/10/2022	Counts	1918	1918	1918	1918	1867	1867
			Min	410	2.00	14.5	− 47.7	11.1	22.7
			Max	602	2.45	− 8.02	− 39.7	33.9	79.1
			Mean	469	2.14	− 10.8	− 41.9	21.2	62.7
			Median	459	2.12	− 10.6	− 41.8	20.3	68.7
			SD	44.6	0.10	1.58	1.15	4.38	14.1

### Meteorological parameters

**Fig. 3:**
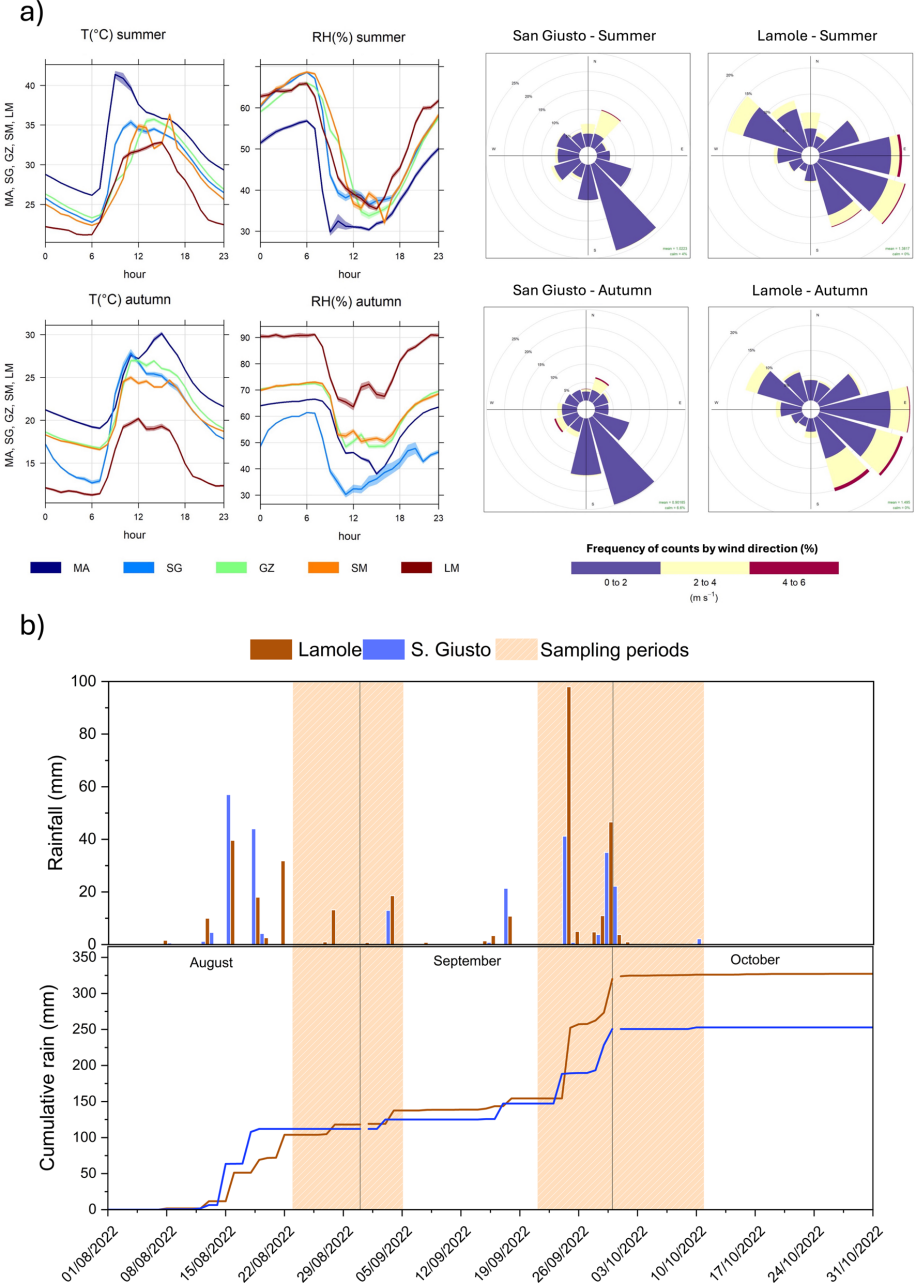
**a** On the left, diurnal cycle of 15-min-averaged air temperature and relative humidity at each monitoring station; on the right, wind roses referred to the two observation periods (summer and autumn), at the two representative meteorological stations, San Giusto and Lamole. **b** Daily cumulative rain amounts, in mm, from August to October 22, recorded at the two selected meteorological stations, and, in the bottom part, monthly cumulative rain amounts (in mm) at two stations

Overall, air temperature was higher in August than in September (Fig. 3a, Tab. S1), reaching the highest average values of 33.3 and 29.0 °C at MA and SG, respectively. The LM site displayed the lowest average temperature (26.1 °C; Tab. S1). A similar daily trend, with maximum values in the early afternoon, was shown in all the measurement sites (Fig. 3a). Relative humidity was higher during the autumn season than in summer, with the highest values recorded at LM (up to 90%; Tab. S1) and showed temporal trends typically opposite to those of temperature (Fig. 3a).

Prevailing wind direction was from SE in the valley (San Giusto meteorological station) and SE-E at the highest latitude (Lamole meteorological station) (Fig. 3a).

August and September were the rainiest months (up to 118 and 202 mm, respectively), whereas October was slightly drier (Fig. 3b). Monthly cumulative rain amounts (in mm) are reported in Fig. 3b (lower part).

### Mobile measurements

The summary descriptive statistical parameters (minimum, maximum, mean, median, and standard deviation) computed on 1-min-averaged data measurements carried out using the mobile monitoring station are reported in Table 2. The first survey (May 26) was performed under sunny weather conditions, with air temperatures ranging from 21 °C (at the beginning of the route) to 31 °C (in the early afternoon hours). The CO_2_ concentrations ranged from 415 to 550 ppm, with δ ^13^C-CO_2_ values from − 23.7 to − 9.36‰ versus V-PDB, while those of CH_4_ were from 1.97 to 2.17 ppm, with δ^13^C-CH_4_ values from − 52.7 to − 39.3‰ versus V-PDB. The second survey (August 23) was carried out under sunny weather conditions, with air temperatures from 24 °C in the early morning, to 33 °C in the middle day. Carbon dioxide ranged from 417 to 620 ppm (δ^13^C-CO_2_ values from − 15.0 to − 8.32‰ vs. V-PDB), while CH_4_ was from 2.01 to 2.17 ppm (δ^13^C-CH_4_ from − 48.1 to − 40.3‰ vs. V-PDB). The third survey (October 25) was performed under cloudy weather and air temperatures varying from 15 °C (around 09:00 a.m.) to 26 °C in the middle hours of the day. Carbon dioxide ranged from 402 to 623 ppm (δ^13^C-CO_2_ values from − 19.1 to − 10.3‰ vs. V-PDB), whereas CH_4_ ranged from 1.98 to 2.38 ppm (δ^13^C-CH_4_ from − 55.1 and − 37.1‰ vs. V-PDB).

**Table 2:** Summary statistics (minimum: Min., maximum: Max., mean, median, and standard deviation: S.D.) of 1-min-averaged CO_2_ and CH_4_ high precision concentrations (in ppm) and isotopic compositions (in ‰ vs. V-PDB) measured during the three transects along the GRb

Monitoring period	CO_2_	∂^13^C-CO_2_	CH_4_	∂^13^C-CH_4_
*May 26*				
Counts	315	315	315	315
Min	415	− 23.7	1.97	− 52.7
Max	550	− 9.36	2.17	− 39.3
Mean	451	− 11.2	2.02	− 43.9
Median	442	− 10.7	2.03	− 43.7
SD	27.5	1.7	0.04	1.92
*August 23*				
Counts	255	255	255	255
Min	417	− 15.0	2.01	− 48.1
Max	620	− 8.32	2.17	− 40.3
Mean	469	− 11.0	2.05	− 43.9
Median	455	− 10.7	2.03	− 43.9
SD	43.6	1.63	0.04	1.33
*October 25*				
Counts	187	187	187	187
Min	402	− 19.1	1.98	− 55.1
Max	623	− 10.3	2.38	− 37.1
Mean	489	− 13.1	2.06	− 45.8
Median	495	− 13.2	2.06	− 44.7
SD	62.7	1.80	0.08	3.46

## Discussion

### Diurnal and seasonal variations at fixed sites

**Fig. 4:**
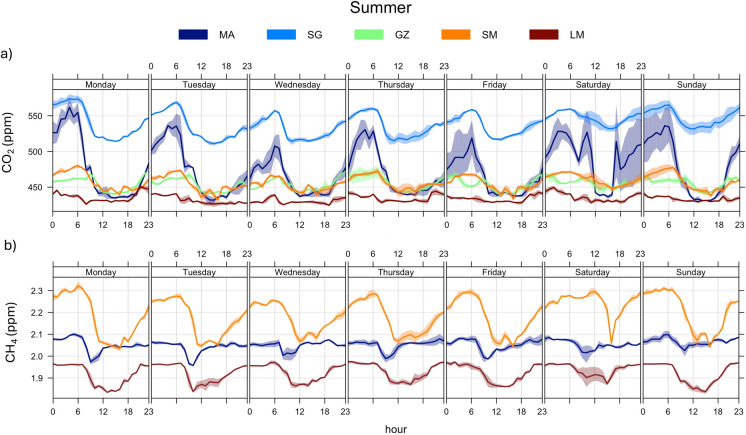
Diurnal cycle and weekly variations during the Summer period (August 2022) of 15-min-averaged **a** CO_2_ concentrations and **b** CH_4_ levels, at five fixed stations

**Fig. 5:**
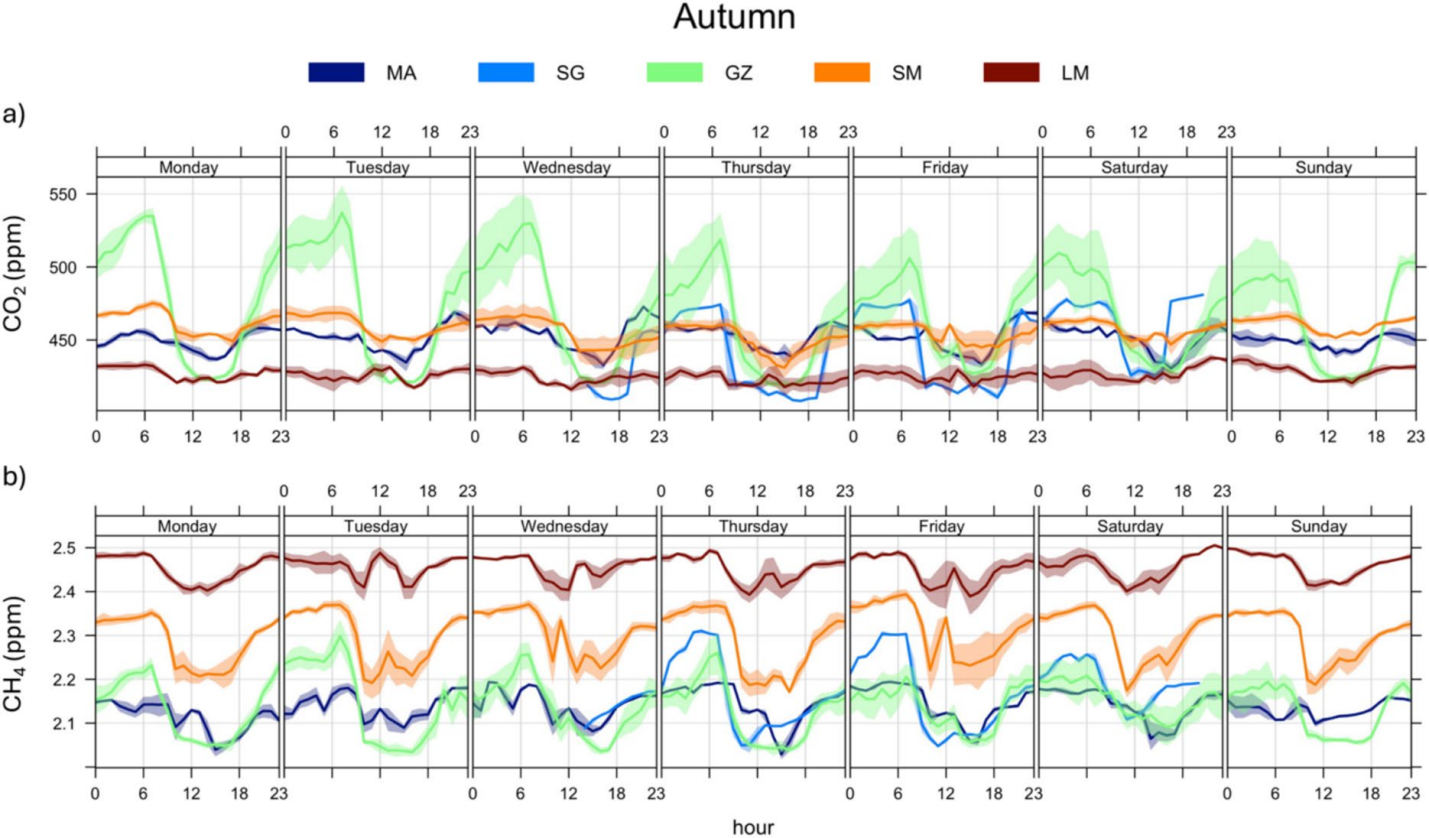
Diurnal cycle and weekly variations during the Autumn period (October 2022) of 15-min-averaged **a** CO_2_ concentrations and **b** CH_4_ levels, at five fixed stations

The daily variation of the CO_2_ and CH_4_ concentrations (diurnal and weekday concentrations) at each fixed monitoring site was controlled by the Planetary Boundary Layer (PBL) (Figs. 4 and 5; diurnal and weekday trends were made with *openair—*R studio package, Carslaw & Ropkins, 2012). The PBL height and its evolution were significantly affected by meteorological conditions, particularly air temperature and wind speed, which in turn were influenced by the day-night cycle. The relatively low wind speed and lack of solar radiation created favorable conditions for the accumulation of GHGs during the nighttime (23:00–05:00; Figs. 4 and 5). Conversely, the enhanced vertical mixing of air strata during the daytime, driven by soil heating, facilitated the dispersion and dilution of CO₂ and CH₄ concentrations (11:00 to 18:00; Figs. 4 and 5). This regulation of the diurnal variability of air pollutants is typical in large cities (Quan et al., 2013, SIR—Regione Toscana, 2022).

The distribution of pollutant concentrations were different in urban and rural zones, respectively, in agreement with previous studies in other areas (Crippa et al., 2021; Hendryx et al., 2010; Pataki et al., 2007; Sharma & Kulshrestha, 2014). In particular, during the summer season (Fig. 4), the highest CO_2_ concentrations were measured at the downstream sites (MA and SG), indicating the occurrence of massive contaminant sources, while at increasing distances from the main urban centers (GZ, SM, and LM) the CO_2_ concentrations strongly decreased (Fig. 4a), settling slightly above background levels (~ 425 ppm; NOAA, 2024). Metropolitan areas typically show a similar spatial distribution of CO_2_ concentrations, being related to the so-called *urban dome* phenomenon (Venturi et al., 2021). However, the MA urban site exhibited lower CO_2_ compared to SG, despite the former being located in closer proximity to the metropolitan center of Florence. This is likely attributable to the relatively low level of anthropogenic activity at MA, which was characterized by a prevalence of small residential buildings and extensive green areas. On average, both urban sites were characterized by relatively large peak-to-through amplitude in the CO_2_ daily cycle (Fig. 4a), likely related to the strength of local emission sources. The MA site turns out to be more influenced by workers’ movements, as evidenced by a peak observed during the morning rush hours (around 06:00) on weekdays, with an anomalous peak during Saturday night when citizens are mostly traveling through the city for recreational purposes (Fig. 4a). At the suburban GZ site, CO_2_ concentrations were lower than those at urban sites, as Galluzzo village is a suburban district not particularly affected by recreational or touristic activities. However, it represents a hub for Florentine worker traffic in and out of the metropolitan city, with both the A1 Milan-Naples highway and the FI-SI highway traversing the area. During this summer monitoring period, many work activities were on break, as testified by the relatively low CO_2_ concentrations measured even during daytime (Fig. 4a). A similar CO_2_ behavior was shown at SM (Fig. 4a). The LM rural site showed the lowest CO_2_ daily peak-to-through amplitude (Fig. 4a), confirming the absence of significant contaminant sources. Nevertheless, the CO_2_ values showed a relatively high variability, probably related to the atmospheric instability typical of hilly areas (e.g., Pataki et al., 2007).

The daily cycle of CH_4_ concentrations showed a similar day-night trend relative to that of CO_2_, with relatively high concentrations from 23:00 to 06:00 (Fig. 4b), when stable atmospheric conditions favored contaminant accumulation in near-surface atmosphere. Surprisingly, the measured CH_4_ concentrations at MA approached the global background (~ 1.90 ppm; NOAA, 2024), indicating that no significant CH_4_ sources occurred in the urban area. Similar CH_4_ concentrations were measured at LM, far from any anthropogenic settlement. Differently, the slightly higher CH_4_ concentrations and the highest daily peak-to-trough amplitude measured at SM suggest the occurrence of a local contaminant source possibly related to the use of agricultural vehicles (Wasilewski et al., 2022) given the widely spread olive tree cultivations.

During the autumn season, the CO_2_ concentrations at MA and SG were lower than in summer (Fig. 2a), while the GZ site exhibited higher concentrations (Fig. 2a). It is worth mentioning that at the MA and SG sites the stations worked for a limited period due to a malfunction. Consequently, the results obtained may not exhaustively represent air quality in that context. No relevant variations concerning the previous summer period were observed at LM and SM (Fig. 2a), confirming the absence of significant emission sources of CO_2_ in the rural and suburban zones. At GZ, wider CO_2_ variations were observed, with the highest peaks associated with morning rush hours (around 07:00) on weekdays and a slightly decreasing trend during the weekend (Fig. 5a). Hence, this hub point was confirmed to be affected by a strong impact of workers’ traffic emissions, particularly evident in the autumn period, when industrial activities in the area were fully operative. On the other hand, the decrease of the CO_2_ concentrations at MA and SG relative to summer was likely due to the main contaminant source of this zone, which was associated with recreational activities typically decreasing during this autumn period. Moreover, at these urban sites, a relatively high atmospheric instability was recorded (with a total rainfall of 140 mm at San Giusto meteorological station and with wind gusts up to 8.4 m/s), which may have favored air cleaning.

The CH_4_ concentrations did not show significant differences comparing the two monitored periods (Fig. 2b), being slightly above the global background concentration (~ 1.90 ppm; NOAA, 2024), except those recorded at the LM site (2.46 ppm on average; Tab. S1). Hence, the lack of significant CH_4_ sources in the urban area was confirmed, whereas at LM, CH_4_ concentrations in the autumn were significantly higher than in the summer (Fig. 2b), possibly related to emissions from organic matter and agricultural activities, similar to what observed at SM, which in the autumn period was the second site for CH_4_ abundance in air (Figs. 2b, 5b). Usually, undisturbed soils act as a net CH_4_ sink but a significant reduction in the CH_4_ oxidation rates occurs when soils are covered by agricultural activities, whose effects have been mainly related to soil disturbance and the ammonium-based N fertilization (Bayer et al., 2012; Hartmann et al., 2011; Suwanwaree & Robertson, 2005; Willison et al., 1995). Noteworthy, in the surroundings of the LM measurement site large arable fields occur (Fig. 1a), where intense agricultural activities are commonly carried out during the autumn. Moreover, CH_4_ oxidation rates are sensitive to temperature variation and rainfall events, which directly influence methanotroph activities and soil water content (Wei et al., 2015; Zhang et al., 2021). During the autumn season, LM experienced the lowest temperatures and the greatest rainfall events (Fig. 3), which may reduce the soil CH_4_ sink strength.

### CO_2_ and CH_4_ sources

**Fig. 6:**
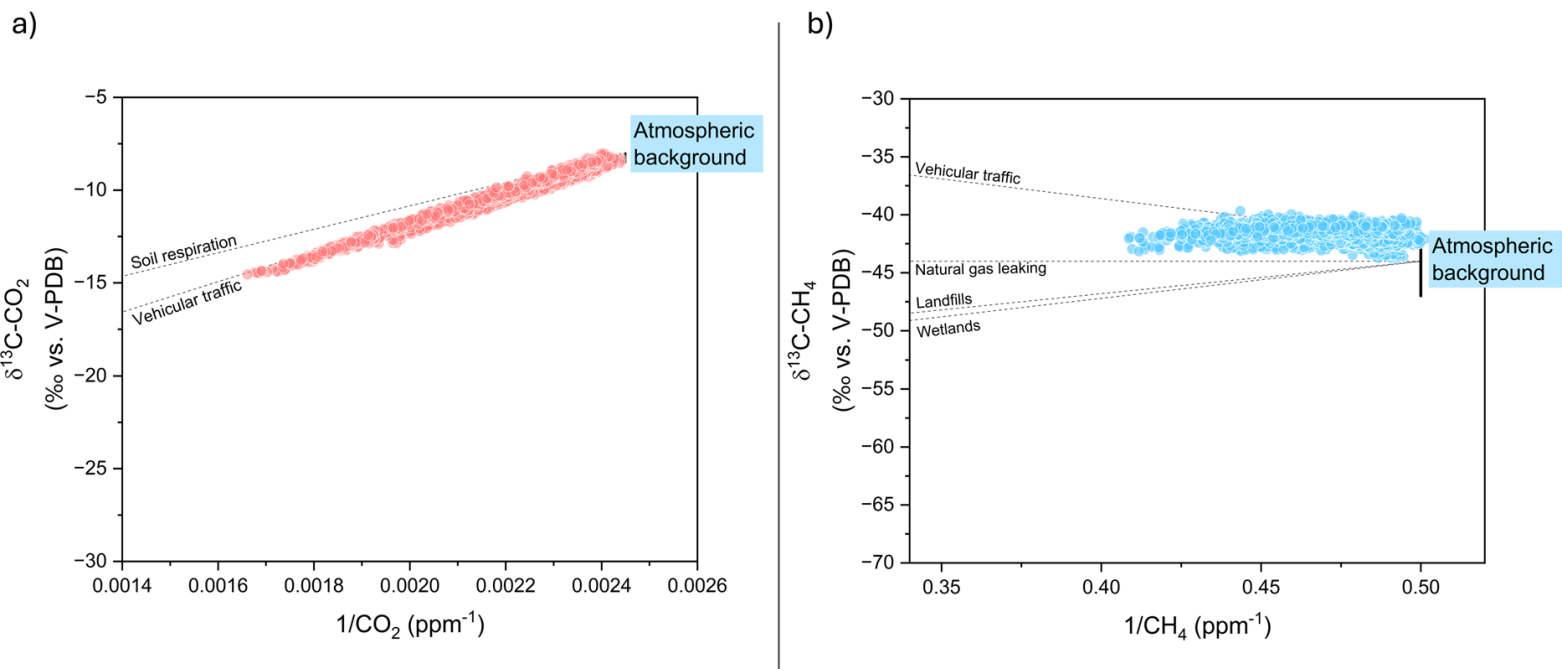
Keeling plot of **a** ∂^13^C-CO_2_ versus 1/CO_2_ and **b** ∂^13^C-CH_4_ versus 1/CH_4_ of 15-min-averaged data recorded at the fixed point GZ, from September 22 to October 11, 2022. The dashed lines show the mixing between background values (expressed as the range between the global clean air value and the minimum one recorded in the study area) and different end members. In particular, we reported CO_2_ vehicular emissions (− 27‰ vs. V-PDB) and soil respiration (− 23.5‰ vs. V-PDB ± 2‰) end members. For CH_4_, we reported vehicular emissions (− 26‰ vs. V-PDB), natural gas leaking (− 44‰ vs. V-PDB), landfills (− 58‰ vs. V-PDB), and wetlands (− 60‰ vs. V-PDB) end members

Sources of air contaminants possibly responsible for the occurrence of anomalous concentrations of CO_2_ and CH_4_ in air in suburban areas, such as in GZ, were tentatively recognized based on the Keeling plot analysis (Keeling, 1958, 1961), using the δ^13^C-CO_2_ and δ^13^C-CH_4_ values measured at GZ by the Picarro analyzer. The Keeling approach assumes that, given a constant background, the isotopic value of the source of a “contaminant” is defined by the intercept of the regression line fitting the points distributed on a binary diagram plotting the measured isotopic values versus the inverse of concentrations of the contaminant (Zazzeri et al., 2017). In the case of air contaminants, the regression line reflects the mixing between the atmospheric background and the potential emission sources, assuming that the background value and gas sources remained constant during the observation period (Venturi et al., 2021, and references therein). For this study, the CO_2_ and CH_4_ Keeling plots report the theoretical mixing lines connecting the background values with those of expected potential sources (Fig. 6a, b). Assuming that the atmospheric background was characterized by δ^13^C-CO_2_ values ranging from − 8‰ to − 8.5‰ versus V-PDB (Venturi et al., 2021) and a 1/CO_2_ ratio of 0.0024 ppm^−1^ (considering 420 ppm as the background CO_2_ concentration), the estimated δ^13^C-CO_2_ intercept of the emitting source was − 28‰ vs. V-PDB (R^2^ = 0.98). This value was consistent with emissions related to vehicular traffic (from − 32 to − 27‰ vs. V-PDB; Venturi et al., 2021) while it is far from the end member related to soil respiration (ranging between − 26.5 and − 20‰ vs. V-PDB; (Górka & Lewicka-Szczebak, 2013) (Fig. 6a), indicating that the former was the main source of CO_2_ in the suburban village of Galluzzo (GZ).

Differently, the measured δ^13^C-CH_4_ values plotted along an alignment between the background value assumed between − 45 and − 47‰ versus V-PDB (Quay et al., 1999) and a 1/CH_4_ ratio of 0.5 ppm^−1^ (considering 2.00 ppm as the local background), and an end member likely deriving from leaking of natural gas from the subterranean pipeline array of the city (from − 40 to − 44‰ vs. V-PDB; Zazzeri et al., 2015) (Fig. 6b), as already suggested for suburban areas similar to GZ in Florence (Venturi et al., 2021; Zazzeri et al., 2015). Therefore, urban traffic (δ^13^C-CH_4_ ranging from − 32 to − 27‰ vs. V-PDB), although representing the main anthropogenic CO_2_ source (Fig. 6a), was confirmed not to significantly contribute to CH_4_ concentrations in air. On the other hand, CH_4_ emissions related to house heating, a classical potential anthropogenic source characterized by δ^13^C-CH_4_ values >  − 25‰ versus V-PDB (Quay et al., 1999; Zazzeri et al., 2017), are to be excluded, as also suggested by the fact that the domestic heating systems were likely off due to the relatively high air temperatures measured during the autumn survey. Likewise, CH_4_ from wetlands (− 60‰ vs. V-PDB; Quay et al., 1999) or from landfills (− 58‰ vs. V-PDB; Zazzeri et al., 2017) is to be excluded as the data do not align towards these end member. This is also supported by the absence of these types of sources around the GZ measurement point.

### Particulate matter

**Fig. 7:**
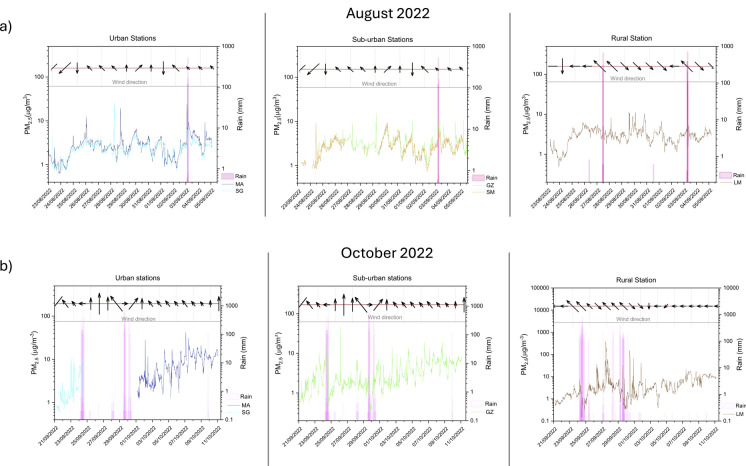
Daily variation of 15-min-averaged PM_2.5_ concentrations during **a** August 2022 and **b** October 2022, at urban (MA and SG points), sub-urban (GZ and SM), and rural (LM) stations. Each plot also shows the main direction and intensity (given by the direction and length of the black arrow, respectively) of the winds, and rainfall events (in mm, pink bars)

The temporal evolution of the PM_2.5_ concentrations measured at the five fixed sites during the two monitored periods (summer and autumn 2022) was compared to the meteorological parameters, i.e. wind direction and speed, and rainfall events (Fig. 7a, b), which typically affect PM formation, transportation, and dilution in air masses (Ouyang et al., 2015). During the summer (Fig. 7a), all sites were affected by rainfall events that occurred days before the sampling period (between August 14 and 18, with a total amount of 101.4 and 39.6 mm at San Giusto and Lamole stations, respectively; Fig. 3b), generating prolonged atmospheric turbulence (with wind gusts up to 22.9 and 15.4 m/S at SG and LM stations, respectively) and resulting in PM concentrations < 5 µg/m^3^ up to August 25. At both urban and sub-urban sites, a slight increase in PM_2.5_ concentrations was recorded from August 26 to 31, likely favored by low winds and the absence of rainfall events. LM showed a similar PM_2.5_ trend relative to the other sites, with some rapid changes in concentrations, likely related to (i) the presence of sporadic PM sources at a local scale and (ii) atmospheric instability typical of relatively high altitudes. At this site, PM_2.5_ peaks were sporadically recorded (from August 30 to September 02), likely tracing air masses from the valley below when the prevailing winds blow from NW (Fig. 7a).

The autumn season (Fig. 7b) was characterized by a higher atmospheric variability relative to the summer, being marked by several rainy days and periods of strong winds, especially during the first half of the monitoring period (from September 21 to October 01, with a total amount of 103.4 and 47.2 mm at San Giusto and Lamole stations, respectively; Fig. 3b). The atmospheric instability experienced by the GRb caused a decrease in PM concentrations throughout the area, as this more chaotic situation favored particulate dispersion. Notwithstanding, the PM_2.5_ concentrations measured since October 01 at the urban (MA) and suburban (GZ) sites exhibited a progressive increase, with levels generally higher than those observed during the summer (with a mean value of 8.09 and 2.44 µg/m^3^ at MA and SG during autumn, while a value of 2.69 and 2.41 µg/m^3^ during summer) and a distinct diurnal pattern. Such a temporal trend was likely due to increasing traffic emissions due to the progressive resumption of work activities after the vacation season, as supported by the relatively high PM_2.5_ concentrations recorded during rush hours, i.e., 07:00–09:00 and 19:00–20:00 (up to 25 µg/m^3^; Fig. 7b). The meteorological stability characterizing these days (between October 01 and 11; Fig. 7b) contributed to enhance PM accumulation within the low atmospheric layer. The rainy days and strong winds characterizing the monitoring period up to October 01 produced a large variability of PM_2.5_ concentrations at LM, too, whereas the stable weather conditions dominating after that date kept PM_2.5_ concentrations below 10 µg/m^3^ (Fig. 7b), confirming the absence of significant PM sources at this rural site.

### Mobile station measurements

**Fig. 8:**
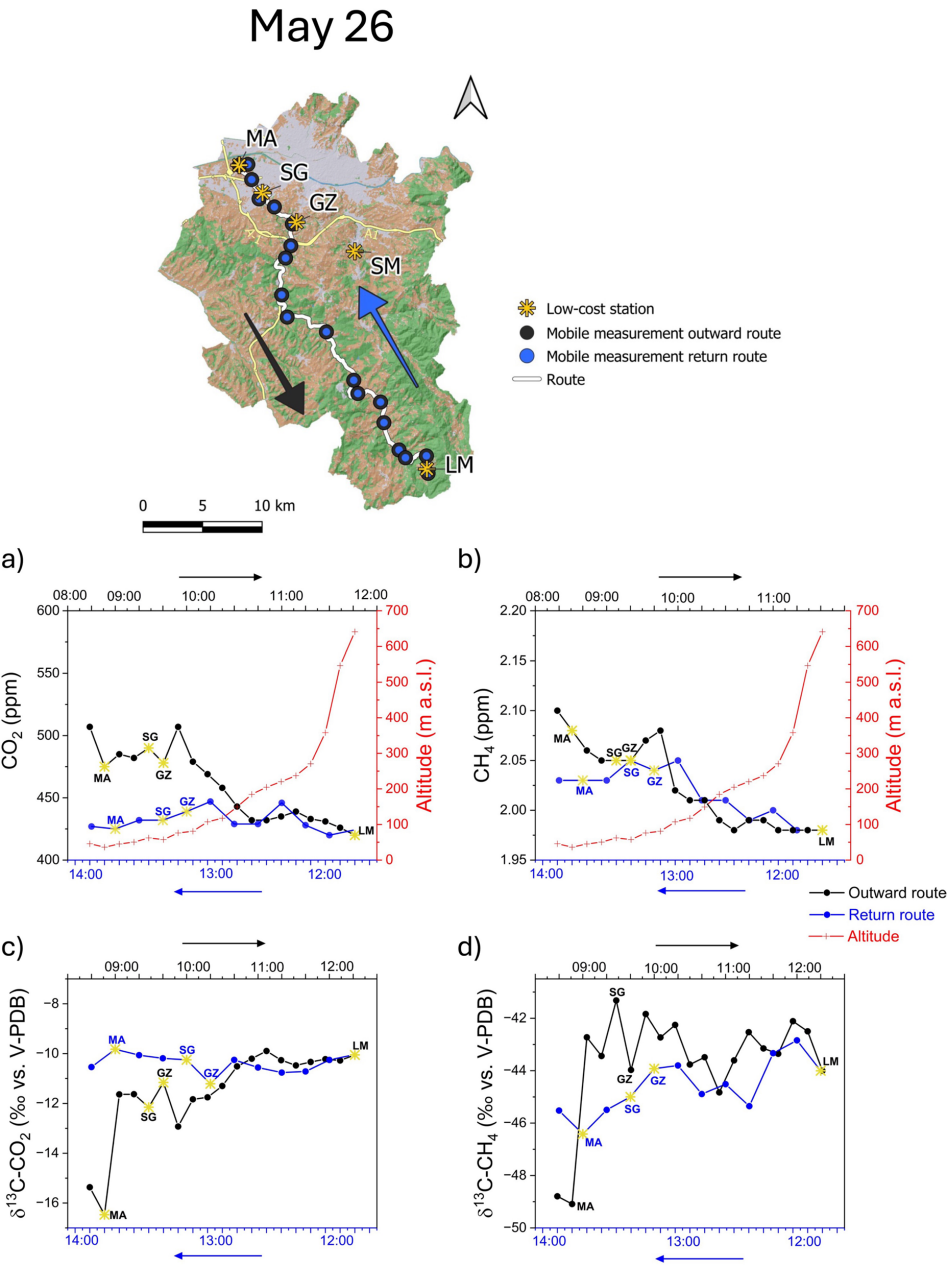
First survey, on May 26, 2022, in the study area (upper map). **a** CO_2_, **b** CH_4_, **c** ∂^13^C-CO_2_, and **d** ∂^13^C-CH_4_ values recorded during the outward route (black line) and the return one (blue line). The graphs also show the five low-cost stations (yellow star), the stop points (black dots), and the elevation (red line). All the data reported are averaged for the time stop (5 min) at each stop point

During the first survey (May 26), relatively high concentrations of CO_2_ and CH_4_ were measured downstream in the early morning (08:00–10:30), while they significantly decreased as the distance from the urban areas increased and moving up in elevation (Fig. 8a,b). On the way back (12:30–14:00) instead, both CO_2_ and CH_4_ concentrations downstream were comparable to those measured upstream, with a significant decrease with respect to the morning levels (Fig. 8a,b). Such changes of air contamination measured at the same sites in different periods of the day were likely related to the PBL evolution, typically characterized by a pronounced atmosphere stratification in the morning followed by vertical mixing in midday, when air gradually warmed up (Quan et al., 2013). The trend of δ^13^C-CO_2_ along the transect provided further corroboration of this evidence, displaying a progressive heavier trend from the downstream urban area (where relatively light values down to − 16.5‰ vs. V-PDB confirmed the prevalence of anthropogenic sources of CO_2_) to the upstream during the morning (up to − 10‰ vs. V-PDB), and almost a steady trend on the way back (Fig. 8c). Noteworthy, the mobile measurements recorded sporadic CO_2_ peaks in distal areas relative to the urban zone (Fig. 8a), likely related to vehicular traffic, such as at the Galluzzo village (close to GZ) where the FI-SI highway is located (Fig. 1a). However, such contamination was relatively low and did not significantly affect the δ^13^C-CO_2_ values of this zone (Fig. 8c). Relatively high CH_4_ concentrations were recorded at the first two measurement points of the survey (Fig. 8b), corresponding with low δ^13^C-CH_4_ values (Fig. 8d), possibly related to biogenic emissions from the Arno River, which flows close to these sites (Fig. 1a). The importance of CH_4_ emission from surface waters was recognized by several authors (Bastviken et al., 2011; Grinham et al., 2018; Holgerson & Raymond, 2016; Stanley et al., 2016) and the potential contribution of urban waterways to greenhouse gas emissions is currently receiving increasing attention. The Arno River receives the combined sewer overflows and wastewater discharges from the city of Florence, resulting in increased nutrient inputs to the river that promote the anaerobic production of CH_4_ (Beaulieu et al., 2020 and references therein).

**Fig. 9:**
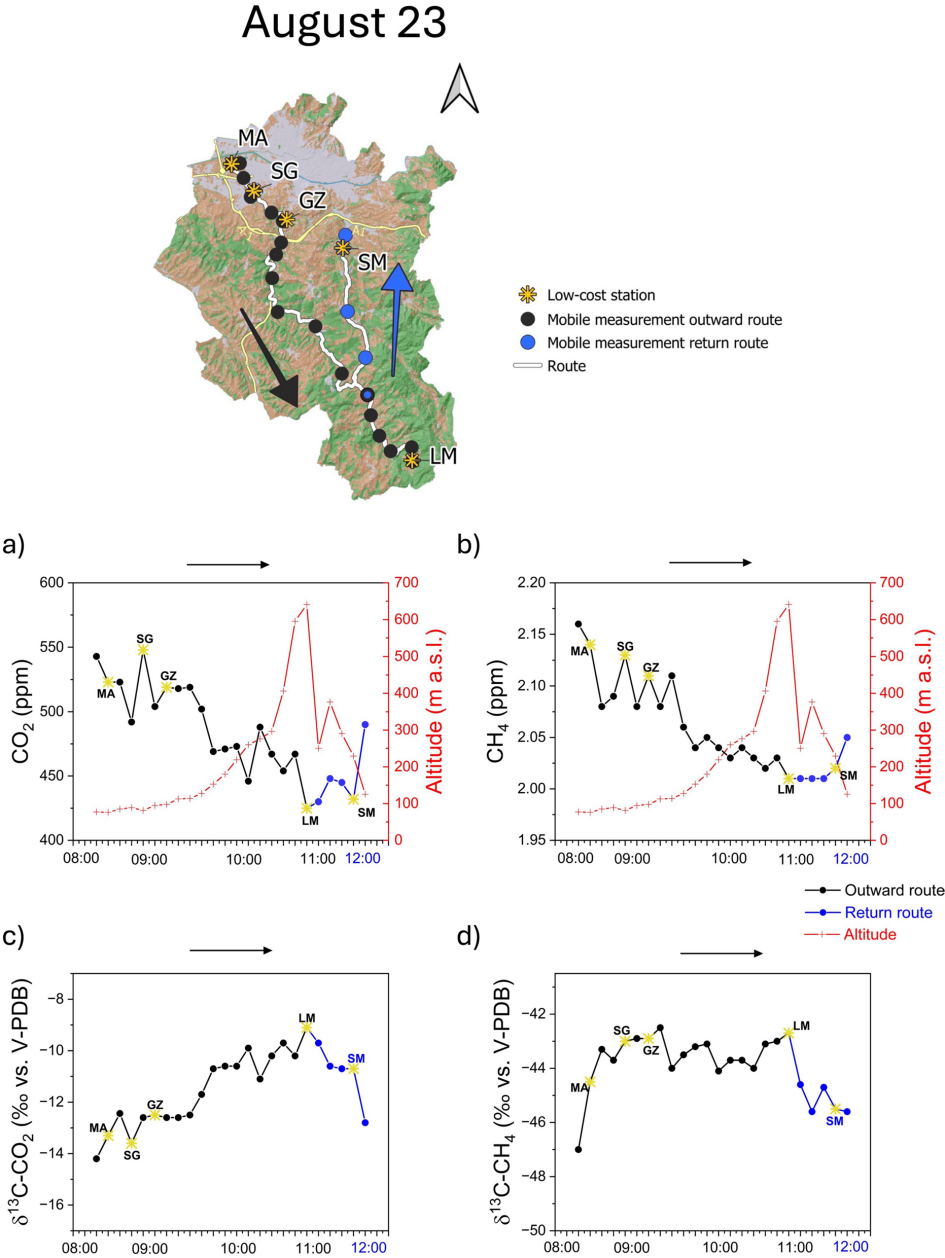
Second survey, on August 23, 2022, in the study area (upper map). **a** CO_2_, **b** CH_4_, **c** ∂^13^C-CO_2_, and **d** ∂^13^C-CH_4_ values recorded during the outward route (black line) and the return one (blue line). The graphs also show the five low-cost stations (yellow star), the stop points (black dots), and the elevation (red line). All the data reported are averaged for the time stop (5 min) at each stop point

The CO_2_ and CH_4_ concentrations measured during a second survey on August 23 (Fig. 9a,b) were basically higher than those recorded in May (Fig. 8a, b), depicting a more pronounced decreasing trend at increasing distances from the city. At the measurement points at relatively high altitudes (≥ 200 m a.s.l), where in May the concentration trends of the two gases were gently decreasing toward the minimum values (Fig. 8a, b), both CO_2_ and CH_4_ showed relatively wide variations (Fig. 9a, b). Such CO_2_ and CH_4_ concentrations peaks recorded in the rural area during the second survey were possibly caused by the intense wind that occurred during the measurements (mean values of 1.4 m/s from the NE at the San Giusto station, and 1.6 m/s from the NW at Lamole station), which likely moved these pollutants from the urban area up to the hills. Vehicular traffic related to tourists that traditionally visit the hilly area during summer may have contributed to the observed CO_2_ peaks. As in May, the δ^13^C-CO_2_ values measured in the downstream urban area during this survey were markedly more negative than in the upstream rural one (Fig. 9c, d), as a consequence of the significant anthropogenic impact. In this case, the concentration peaks recorded in some points far from the city (Fig. 9a, b) also correspond to significant variations of the CO_2_ isotopic values (Fig. 9c). This confirms that air contamination also affected the rural area during this survey. The distribution of the δ^13^C-CH_4_ values seemed to be not strictly related to that of the gas concentration, except at the starting point of the survey where the highest CH_4_ concentration corresponded to the lowest δ^13^C-CH_4_ value being likely influenced, as for the previous survey, by the proximity of the Arno River (Fig. 9d).

**Fig. 10:**
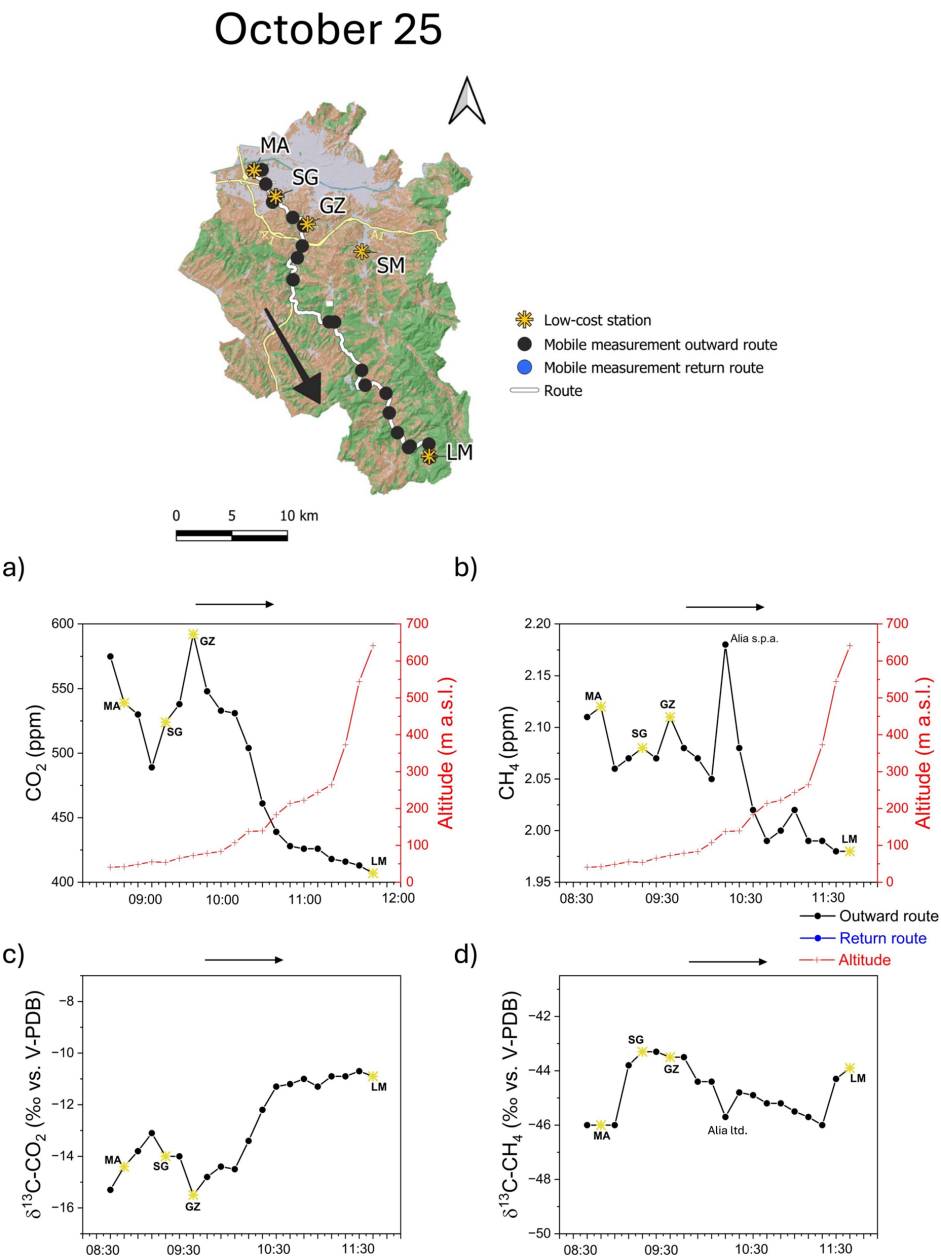
Third survey, on October 25, 2022, in the study area (upper map). **a** CO_2_, **b** CH_4_, **c** ∂^13^C-CO_2_, and **d** ∂^13^C-CH_4_ values recorded during the outward route (black line). The graphs also show the five low-cost stations (yellow star), the stop points (black dots), and the elevation (red line). All the data reported are averaged for the time stop (5 min) at each stop point

During the third survey (October 25), the highest CO_2_ concentrations were recorded at the starting point (in the urban area of Florence) and at GZ. Overall, CO_2_ levels remained relatively high until the altitude overcame 200 m a.s.l., when they strongly decreased down to a minimum value at LM (Fig. 10a). Such a spatial–temporal pattern was likely dictated by the occurrence of extensive cloud cover and low winds measured at the urban San Giusto station (0.6 m/s from SE; SIR, 2022), which favored the maintenance of atmospheric stratification established during the night and early morning, limiting the dilution of air contaminants accumulated in the valley. More intense winds were recorded at the Lamole station (2.9 m/s SE; SIR, 2022), where the lowest CO_2_ concentrations were measured (407 ppm), being likely related to clear air from the surrounding hilly areas. These hypotheses were supported by CH_4_ concentrations (Fig. 10b) that followed those of CO_2_, except a peak recorded close to an area used for urban waste collection (Alia Ltd.), which may have represented a CH_4_ source (Sadegh Sekhavatjou et al., 2012). As for the previous surveys, relatively low δ^13^C-CO_2_ values corresponded to increased CO_2_ concentrations (Fig. 10c). Relatively low δ^13^C-CH_4_ values marked those sites where the CH_4_ concentrations were influenced by the Arno River (first two points of the survey) and emission from the exposed waste (δ^13^C-CH_4_ values from − 90 to − 45‰ vs. V-PDB; Bakkaloglu et al., 2022). Differently, the concentration peak recorded at GZ (Fig. 10b) was not accompanied by significant changes of the δ^13^C-CH_4_ value (Fig. 10d).

**Fig. 11:**
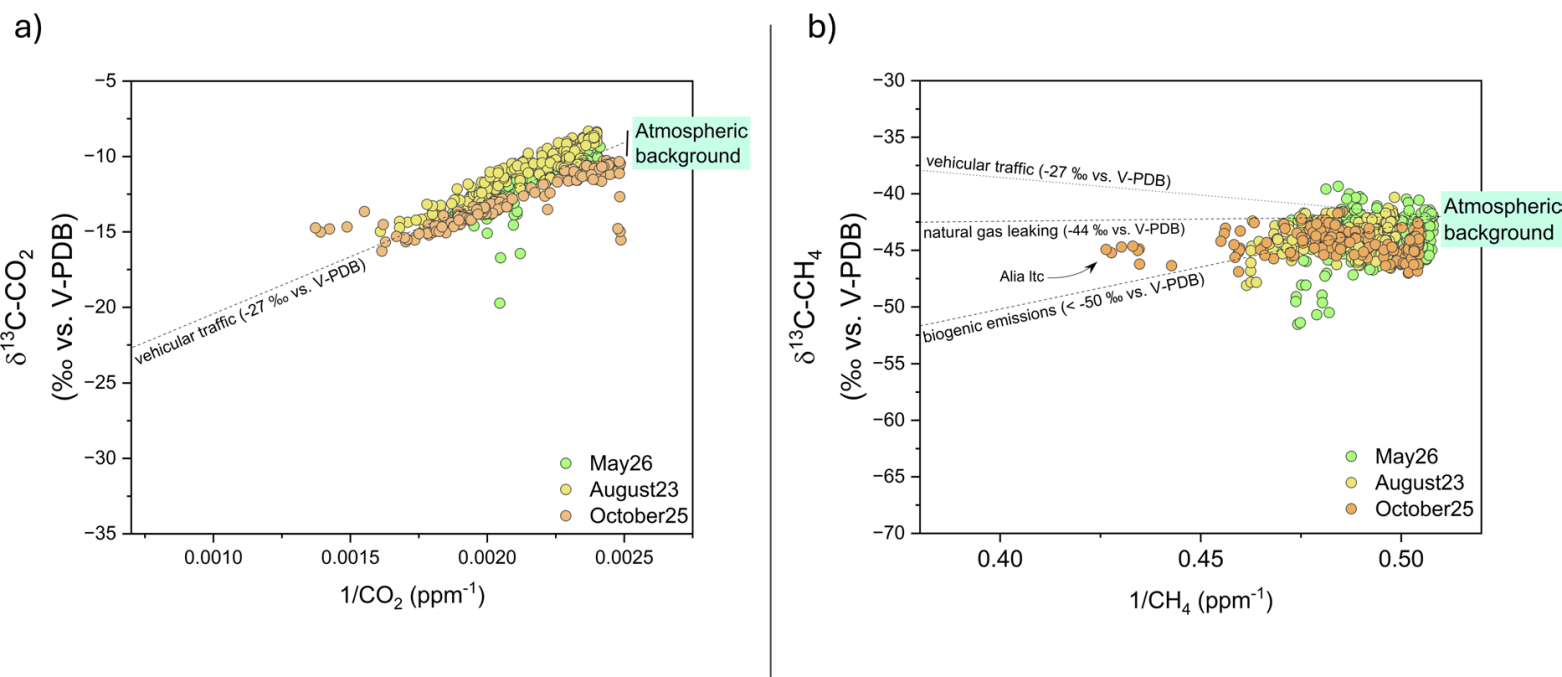
Keeling plot of **a** ∂^13^C-CO_2_ versus 1/CO_2_ and **b** ∂^13^C-CH_4_ versus 1/CH_4_ of 1-min-averaged data recorded during the three mobile measurements (May, August, and October 22). The dashed lines show the mixing between background values and vehicular emissions endmembers (in **a**), and between background and vehicular emissions, natural gas leaks, and biogenic emissions endmembers (in **b**)

Vehicular traffic as the main anthropogenic CO_2_ source affecting air during the three surveys carried out with the mobile station was also confirmed by the Keeling plot analysis (Fig. 11a). Methane isotopic values rule out a predominant source of this species but show a mixture of sources, as evidenced by the intermediate δ^13^C-CH_4_ values ranging between biogenic emissions, typically displaying δ^13^C-CH_4_ values <  − 50‰ versus V-PDB (Zazzeri et al., 2017), and leakage from pipelines, generally with values from − 40 to − 44‰ versus V-PDB (Fig. 11b).

## Conclusions

A traditional air monitoring array, commonly based on few expensive monitoring stations located at fixed sites, can provide data with high accuracy and precision, but is affected by a low spatial resolution. In this study, the highly variable distribution of the air contaminants in the southern sector of Florence city and its surroundings was investigated by combining two approaches, as follows: (i) measurements of CO_2_, CH_4_, and PM_2.5_ concentrations using a dense network of low-cost fixed stations positioned within the study area, to monitor the temporal variation of pollutants in strategic sites thought to represent different situations, e.g. rural zones, urban and suburban settlements; (ii) measurements of CO_2_ and CH_4_ concentrations in air and their ^13^C/^12^C ratios using a mobile station, recording a single snapshot of air pollutant at a series of locations at specific time frames. Data measured by both the mobile and fixed stations consistently indicated that the urban area was significantly affected by anthropogenic emissions mainly associated with vehicular traffic, while rural areas, often characterized by greater atmospheric instability and showing, with few exceptions, no significant contaminant sources, were scarcely polluted. Rainfall events, wind speed and direction, as well as air temperature, controlled the evolution of PBL, which had a key role in the spatial and temporal distribution of the air contaminants in the study area, characterized by drastic differences in elevation.

Overall, the results of this study emphasize the limitations of the classical approach carried out using high-performances and expensive instruments placed at fixed stations, which, despite their high sensitivity and accuracy, fail to provide complete coverage of highly heterogeneous urban environments. In contrast, low-cost instruments allow to develop an efficient, high-resolution monitoring network, able to provide a comprehensive assessment of atmospheric pollutant distribution. Moreover, this study demonstrated that the adoption of combined measurement methods, i.e. using both fixed and mobile stations, is highly recommended to provide an exhaustive description of air contaminant distribution in contaminated areas at a broad spatial and temporal scale.

## Supplementary Information

Below is the link to the electronic supplementary material.

Supplementary file1 (PDF 5093 KB)

Supplementary file2 (PDF 1428 KB)

Supplementary file3 (PDF 530 KB)

Table S1 Summary statistics (minimum: Min., maximum: Max., mean, median, and standard deviation: S.D.) of 15-minute-averaged CO_2_ and CH_4_ concentrations (in ppm), PM2.5 (in µg/m-3), temperature (in °C) and relative humidity (in %) values at each monitoring station, during the two sampling periods (Summer and Autumn 2022). (XLSX 14 KB)

## Data Availability

No datasets were generated or analysed during the current study.
