# Supplementary material for: Traditional and low-cost technical approaches for investigating greenhouse gases and particulate matter distribution along an urban-to-rural transect (Greve River Basin, Central Italy)
Source: Environ Geochem Health. 2025 Mar 27;47(5):138. doi: 10.1007/s10653-025-02456-2 (PMC11946975; doi:10.1007/s10653-025-02456-2)
Supplement: Supplementary file 1 — Supplementary file1 (PDF 5093 KB) [file 10653_2025_2456_MOESM1_ESM.pdf]

Some photos of the five multiparametric low-cost station locations: Mantignano (MA), San Giusto (SG), Galluzzo (GZ), San Martino (SM), and Lamole (LM)

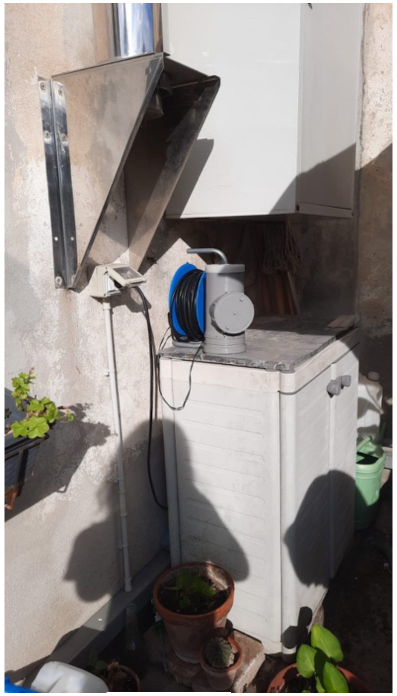

MA

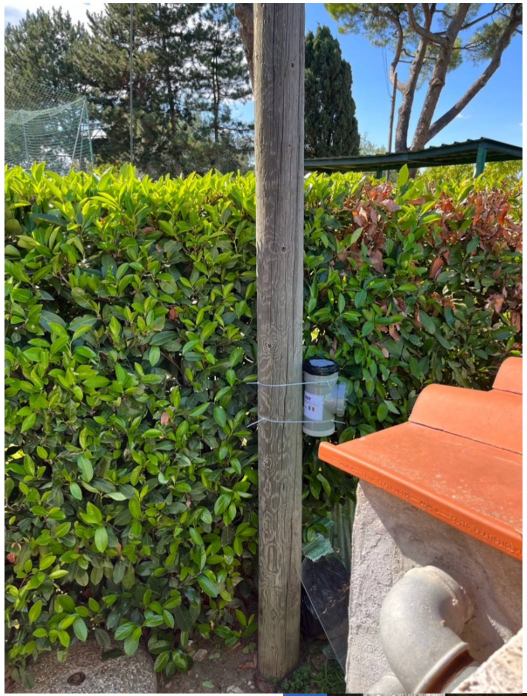

SG

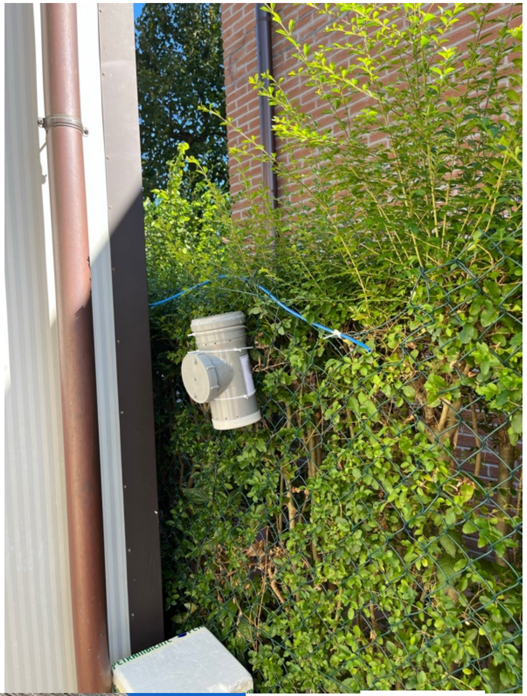

GZ

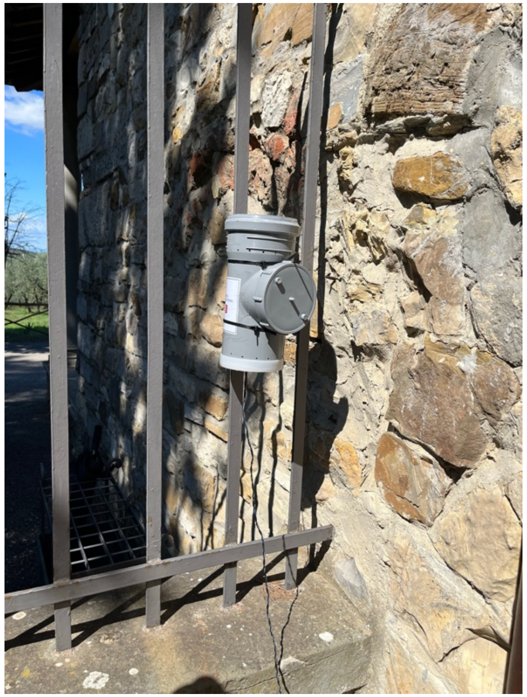

SM

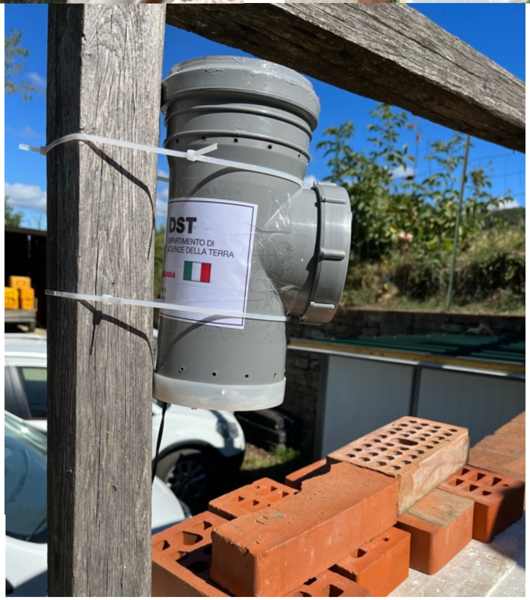

LM
