# Supplementary material for: Traditional and low-cost technical approaches for investigating greenhouse gases and particulate matter distribution along an urban-to-rural transect (Greve River Basin, Central Italy)
Source: Environ Geochem Health. 2025 Mar 27;47(5):138. doi: 10.1007/s10653-025-02456-2 (PMC11946975; doi:10.1007/s10653-025-02456-2)
Supplement: Supplementary file 2 — Supplementary file2 (PDF 1428 KB) [file 10653_2025_2456_MOESM2_ESM.pdf]

a)

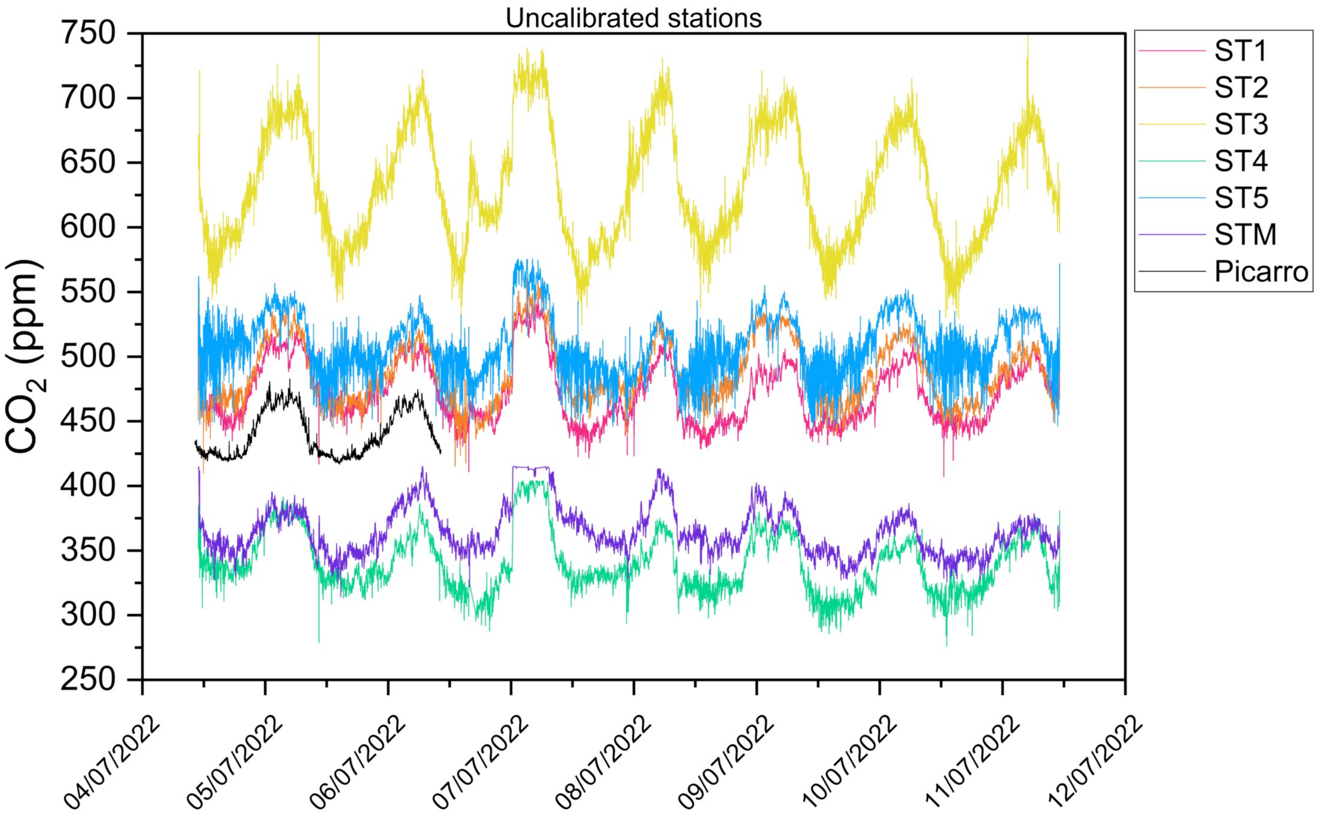

b)

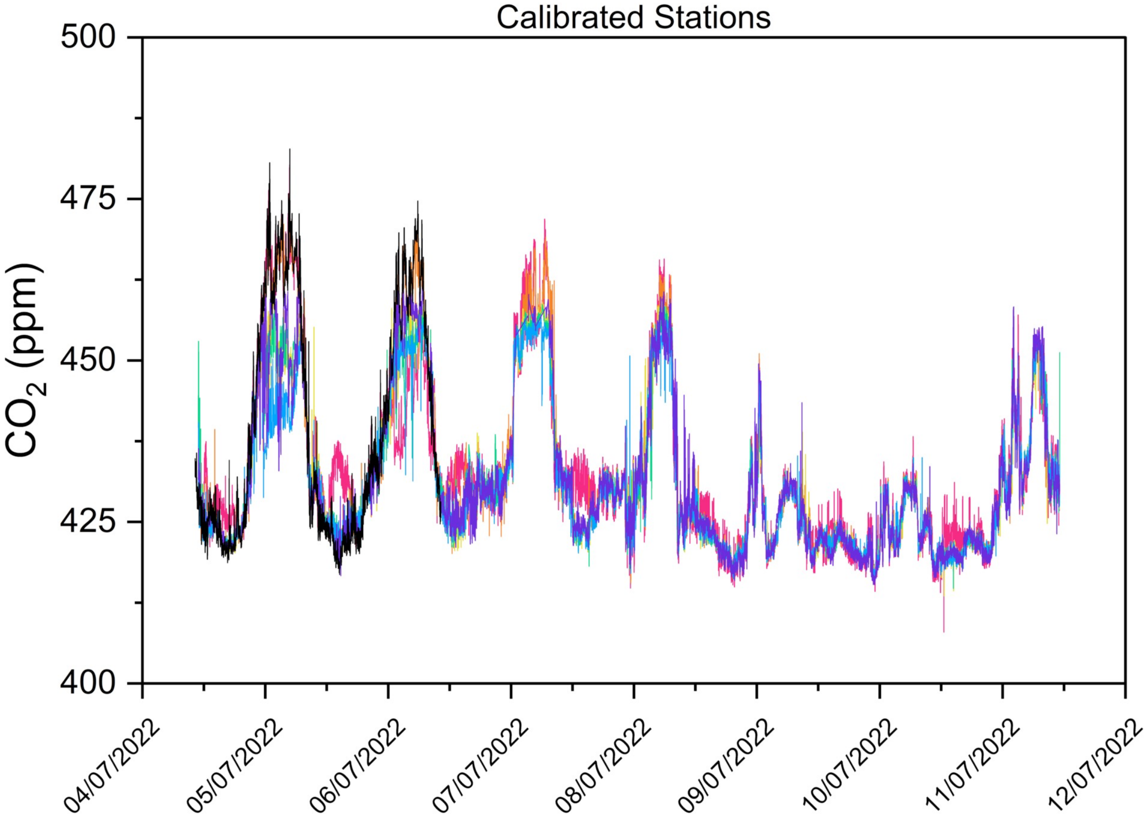

S1. (a) CO_2 raw data (in ppm) of the five low-cost sensors (colored lines) and Picarro measurements (in ppm, black line) recorded at SG point, from July 04 to 11, 2022, used to build the calibration model. (b) calibrated CO_2 concentrations (in ppm) after the processing in the calibration model.

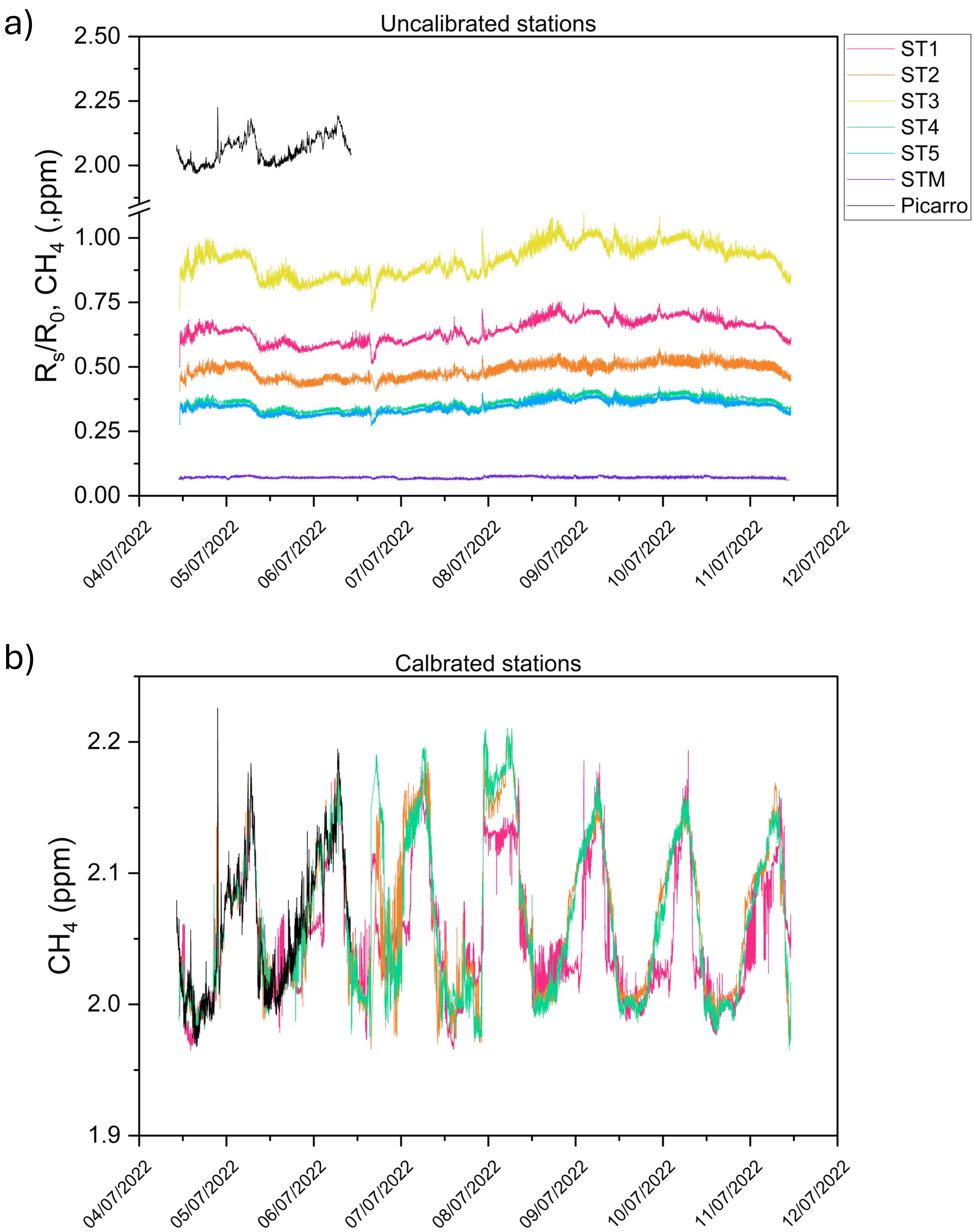

S1. (a) CH_4 raw data (Rs/R0) of the five low-cost sensors (colored lines) and Picarro measurements (in ppm, black line) recorded at SG point, from July 04 to 11, 2022, used to build the calibration model. (b) calibrated CH_4 concentrations (in ppm) after the processing in the calibration model.
