# Supplementary material for: Traditional and low-cost technical approaches for investigating greenhouse gases and particulate matter distribution along an urban-to-rural transect (Greve River Basin, Central Italy)
Source: Environ Geochem Health. 2025 Mar 27;47(5):138. doi: 10.1007/s10653-025-02456-2 (PMC11946975; doi:10.1007/s10653-025-02456-2)
Supplement: Supplementary file 3 — Supplementary file3 (PDF 530 KB) [file 10653_2025_2456_MOESM3_ESM.pdf]

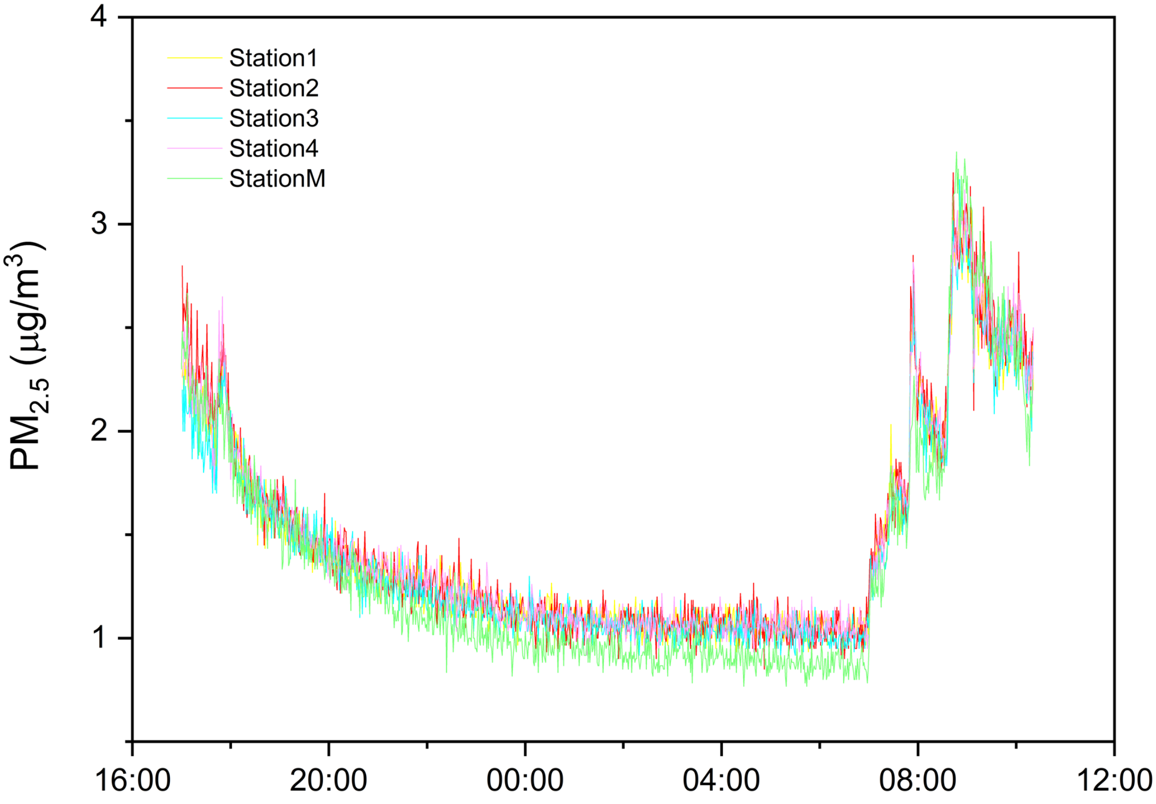

	$PM_{2.5}$		
	SD	Coefficient of Variation	Mean
Station1	0.50169	0.34597	1.45009
Station2	0.53895	0.36614	1.472
Station3	0.4992	0.35084	1.42286
Station4	0.52394	0.35649	1.46973
StationM	0.58318	0.4307	1.35403

S2. Repeatability test of the PM sensor signals, evaluated over a work time of more than 12 hours. The table reports descriptive statistical parameters (standard deviation, coefficient of variation, and mean value)of the five-station outputs.
